# Multidisciplinary Design Optimization of a Novel Sandwich Beam-Based Adaptive Tuned Vibration Absorber Featuring Magnetorheological Elastomer

**DOI:** 10.3390/ma13102261

**Published:** 2020-05-14

**Authors:** Mostafa Asadi Khanouki, Ramin Sedaghati, Masoud Hemmatian

**Affiliations:** Department of Mechanical, Industrial and Aerospace Engineering, Concordia University, Montreal, QC H3G 1M8, Canada; mostafa.asadi@concordia.ca (M.A.K.); masoud.hemmatian@concordia.ca (M.H.)

**Keywords:** multidisciplinary design optimization, magnetorheological elastomers, adaptive tuned vibration absorber, smart sandwich beam

## Abstract

The present study aims to investigate the dynamic performance and design optimization of a novel magnetorheological elastomer based adaptive tuned vibration absorber (MRE-ATVA). The proposed MRE-ATVA consists of a light-weight sandwich beam treated with an MRE core layer and two electromagnets installed at both free ends. Three different design configurations for electromagnets are proposed. The finite element (FE) model of the proposed MRE-ATVA and magnetic model of the electromagnets are developed and combined to evaluate the frequency range of the absorber under varying magnetic field intensity. The results of the developed model are validated in multiple stages with available analytical and simulation data. A multidisciplinary design optimization strategy has been formulated to maximize the frequency range of the proposed MRE-based ATVA while respecting constraints of weight, size, mechanical stress, and sandwich beam deflection. The optimal solution is obtained and compared for the three proposed ATVA configurations. The optimal ATVA with a U-shaped electromagnet shows more than 40% increase in the natural frequency while having a total mass of 596 g.

## 1. Introduction

Magnetorheological elastomer (MRE) is a functional material which responds to an applied magnetic field by changing its viscoelastic properties. MREs are basically the solid analog of magnetorheological fluids (MRFs) as they are composed of micron-sized ferromagnetic particles, typically carbonyl iron powder dispersed into a nonmagnetic solid polymeric or elastomeric medium such as silicone rubber. MREs overcome the problems that accompany the applications of magnetorheological (MR) fluids, such as sedimentation of particles, sealing issues, and environmental contamination. The magnetic properties of MREs originate from microstructural interactions under a magnetic field and depend on different parameters such as volume fraction and spatial distribution of embedded ferromagnetic particles [1,2,3]. When exposed to an external magnetic field, MRE changes its dynamic properties, including both stiffness and damping, rapidly and reversibly, thus, making it an ideal candidate for the development of the next generation of adaptive devices with controllable operating frequency and damping [4].

One intriguing application of MREs is in the development of adaptive vibration absorbers which can be effectively utilized to attenuate vibration in a broad range of frequencies. To date, several designs have been proposed in the literature for vibration absorbers featuring MREs. Lerner and Cunefare [5] designed three types of MRE-based vibration absorbers working in shear, squeeze, and longitudinal modes, and among these, the squeeze mode absorber exhibited the widest frequency range. Sun et al. [6] designed and prototyped a squeeze working mode MRE-based absorber that demonstrated a frequency range of 37 to 67 Hz by providing magnetic field intensity ranging from 0 to 250 kA/m. There are also other important research studies that have investigated the application of MREs in adaptive vibration absorbers [7,8,9]; however, one important task in the development of efficient MRE-based absorbers is the optimization of these smart devices to achieve the best performance.

There have been efforts that considered the analysis and optimal design of MR dampers and absorbers. Parlak et al. [10] carried out optimization on an MR damper’s geometry pursuing two objectives of target damper force and maximum magnetic flux density. They used finite element-based electromagnetic and CFD tools of ANSYS to perform the optimization. Nguyen and Choi [11] proposed an optimal design for a passenger vehicle MR damper which was constrained in a specific volume. The objective function involved damping force, the dynamic range, and inductive time constant of the damper. In recent years, there have also been a number of studies that investigated the design optimization of rotary and translational MR-based dampers and absorbers [12,13,14]. Nevertheless, research studies that analyze MR vibration absorber devices which have continuous structures such as beams are very limited. Particularly, there have been no study conducted on the development of design optimization strategies for such adaptive devices. Hirunyapruk et al. [15] proposed a three-layer beam-like tuned vibration absorber which was treated with MRF in the core layer. Two electromagnets were considered on the beam to apply a magnetic field on the MRF layer. One unique feature of the sandwich beam with treated MR fluid core layers is that the stiffness of the beam and, subsequently, its natural frequencies can be continuously changed by activating the MR fluid. Hirunyapruk et al. demonstrated that the natural frequency of the device could change from 106 to 149 Hz [15]. While this work is very useful for demonstrating the feasibility and operation of beam-like absorber devices, the optimal design problem of the absorber has not been addressed. To the best of our knowledge, there have been no study conducted on the development and multidisciplinary design optimization of adaptive vibration absorbers featuring multilayer MRE-based sandwich structures.

Consequently, the main contribution of this work is, first, to propose three different novel configurations for an MRE-based sandwich beam type adaptive tuned vibration absorber (MRE-ATVA) and, then, to propose a multidisciplinary optimization framework to maximize their frequency bandwidth. To this end, the comprehensive models of the proposed MRE-ATVAs are developed which consists of the following two components: the finite element (FE) model of the sandwich beam, and the mathematical formulation for magnetic analysis of the electromagnets. The MRE in the core layer of the sandwich beam operates in shear mode, and thus its shear modulus changes under the applied magnetic field generated by the electromagnets installed at the tips of the beam. Thus, the natural frequency of the absorber can be continuously varied and controlled through the application of the required magnetic field. The electromagnets on the beam ends play a two-fold role by providing the required magnetic field on the MRE layer, as well as oscillating as the absorber mass. Three different design configurations are considered for the electromagnets to have insights on the flexible design of the proposed absorber device. Then, using the developed model of the absorber, a multidisciplinary optimal design problem which considers both the structural geometry and electromagnet parameters as design variables, is formulated aiming at maximizing the frequency range of the absorber, respecting constraints on the total mass, static deflection, and maximum stress in the beam. To accurately identify the optimal design, the optimization problem is solved using a combination of genetic algorithm (GA) and powerful sequential quadratic programming (SQP) methods. Then, the identified optimal designs for the proposed MRE-ATVAs are compared and discussed.

In this paper, first, the configuration of the proposed MRE-ATVAs with three potential designs of the electromagnet are introduced. Secondly, the FE model of the sandwich beam and magnetic model of the electromagnets are developed and validated. By integrating the FE model of the beam with the magnetic model of the electromagnets, the high-fidelity model of the absorber device is obtained which is subsequently utilized for the development of the optimization problem. Finally, the multidisciplinary optimization problem is formulated considering both structural and magnetic parameters as design variables aiming at maximizing the frequency bandwidth of the proposed MRE-based ATVAs under constraints of mass, size, beam deflection and maximum stress. The optimization results are obtained using the combination of a stochastic based genetic algorithm and gradient-based SQP programming. Then, the relative performances of the optimal ATVAs are compared and discussed with respect to the frequency range and final mass of the device.

## 2. Configuration of the Proposed MRE-Based ATVAs

**Figure 1:**
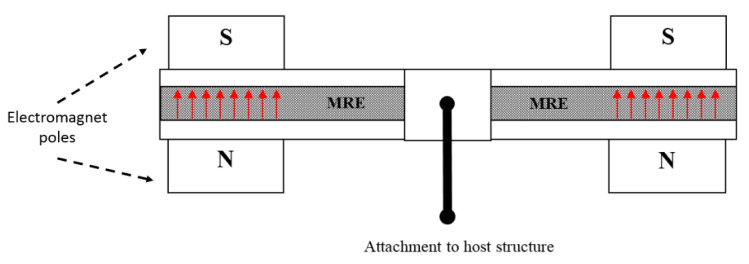
Schematic design of the proposed magnetorheological elastomer (MRE)-based sandwich beam type adaptive tuned vibration absorber.

The main components of the proposed MRE-ATVA are a three-layer sandwich beam and two electromagnets attached at both ends. The sandwich beam consists of an MRE core layer which is constrained between two thin steel layers. The MRE-based sandwich beam provides a unique opportunity to provide distributed control force through variation in the stiffness and damping of the MRE layer. Two electromagnets are installed on both ends of the sandwich beam which provide the required magnetic field for the activation of the MRE, and also act as the absorber’s active mass. Moreover, the location of the electromagnet can change across the length of the beam which can enhance the authority of the proposed MRE-ATVA for different frequency ranges. The schematic design of the proposed MRE-ATVA is illustrated in Figure 1. The MRE core layer is under shear deformation, while the magnetic flux lines, shown by arrows in Figure 1, are perpendicular to the shear direction. The shear stiffness of the MRE is adjusted by varying the magnetic field through changing the current supplied to the coils of the electromagnets. Hence, the stiffness and subsequently the natural frequency of the absorber can be tuned to the desired frequency. The proposed MRE-ATVA can be attached from its middle point to the host system to mitigate vibration within the operating frequency range.

**Figure 2:**
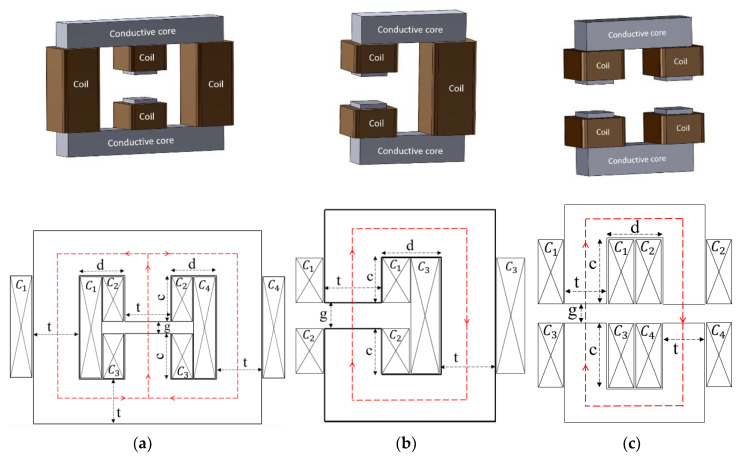
Three electromagnet designs considered for the proposed adaptive tuned vibration absorber (ATVA). (**a**) H-shaped electromagnet; (**b**) C-shaped electromagnet; and (**c**) U-shaped electromagnet.

Three different shapes including the H-, C-, and U-shaped designs are considered for the electromagnets in the present study. In-plane (2D) and in-space (3D) sketches of the proposed design configuration for electromagnets are illustrated in Figure 2. The characteristics and performance of each design could be different in terms of the induced magnetic flux density in the gap and mass of the electromagnet.

As shown in Figure 2, the H-shaped electromagnet has four coils of two different sizes. The C-shaped electromagnet has three coils and two of the coils are of the same size. The U-shaped electromagnet has four similar coils. The depth of the conductive core is the same as thickness t. Therefore, the conductive core of the electromagnets has a square cross-section with an edge size of t. The path of magnetic flux in the core of each electromagnet is shown with a dashed line.

**Figure 3:**
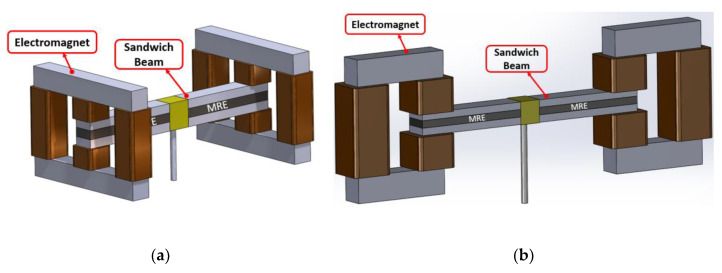

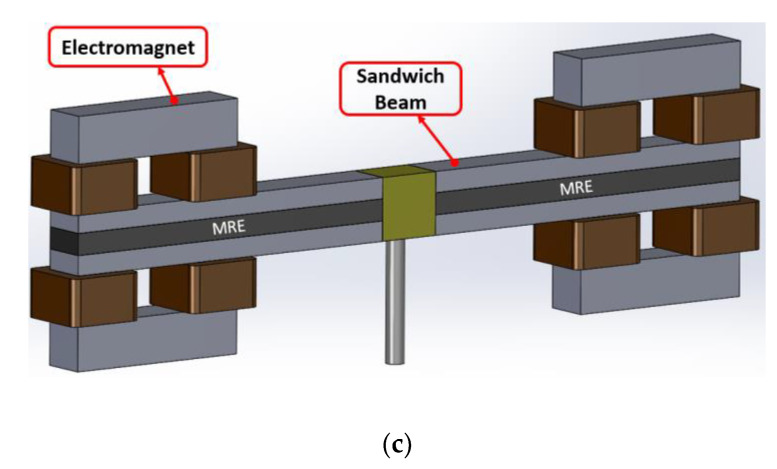
Schematics of the assembled ATVA structure with each of the three electromagnet designs. (**a**) H-shaped electromagnet; (**b**) C-shaped electromagnet; and (**c**) U-shaped electromagnet.

The schematics of the assembled structure of the MRE-ATVA with each of the three electromagnet configurations are also shown in Figure 3. From the figure, it can be realized that the proposed MRE-ATVAs are symmetric from the center of the sandwich beam, and thus behave similarly to the two connected cantilever beams. Therefore, half of the MRE-ATVA, which is a cantilever sandwich beam with one electromagnet attached at the free end, is considered for the optimization problem in the modeling and optimization formulation.

## 3. Mechanical and Magnetic Properties of Materials

**Figure 4:**
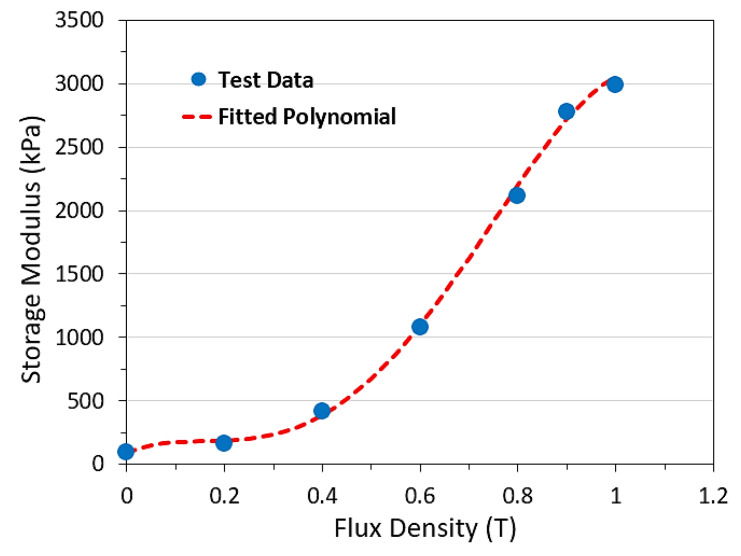
Storage modulus in terms of flux density for the selected MRE sample.

In this section, the mechanical and magnetic properties of the MRE and steel materials which are used as the core and surface layers of the sandwich beam, respectively, are provided. These material properties are utilized in the modeling and design optimization of the MRE-ATVA in the next sections. An MRE sample with 40% volume fraction of iron particles is considered and its properties are adopted from our previous work [1]. This MRE sample was fabricated using silicone rubber as the elastomeric matrix material and soft type ferromagnetic carbonyl iron particles as the magnetic fillers. The selected silicone rubber has low viscosity which facilitates the distribution of magnetic fillers in the matrix and ensures easy mixing and degassing. Details of the fabrication and testing of the MRE sample are explained in [1]. The density of the MRE sample is 3500 kg/m^3^. Figure 4 shows the storage modulus ($G^{\prime }$) in terms of magnetic flux density (B) for the selected MRE material taken from the experimental characterization tests.

A fourth-order polynomial is fitted to the experiment data which is shown by a dashed line in Figure 4 and can be expressed as:

(1) $$G^{\prime }=-1.36\times 10^{7}\;B^{4}+2.47\times 10^{7}\;B^{3}-9.66\times 10^{6}\;B^{2}+1.52\times 10^{6}\;B+9.35\times 10^{4}\;0\leq B\leq 1\;T$$

where $G^{\prime }$ is in kPa and *B* in Tesla (T). It is assumed that for flux density values more than 1.0 T, the MRE sample becomes magnetically saturated and so the storage modulus remains constant and equal to its value at 1.0 T of magnetic field.

**Figure 5:**
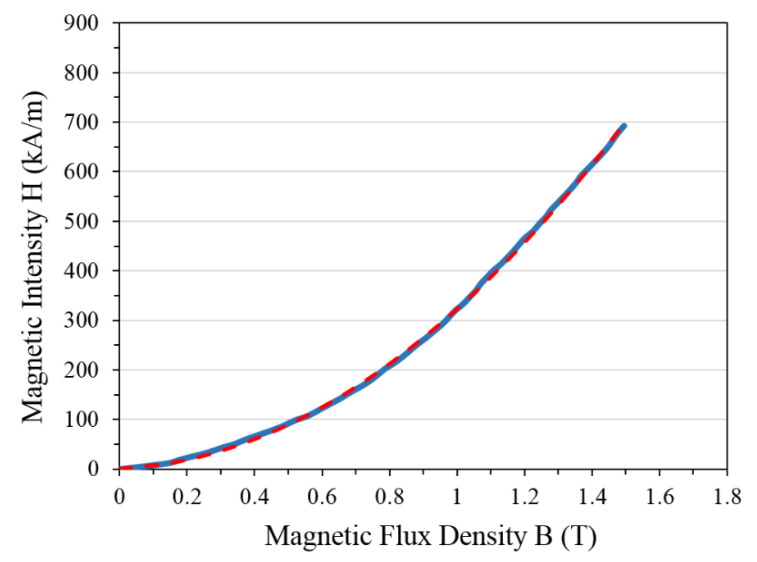
The B–H curve of the MRF-132DG from Lord Corporation [16].

In addition to the mechanical properties, magnetic characteristics are also required for modeling and optimization of the proposed MRE-ATVA. Considering that MR elastomer and MR fluid both have similar mechanisms for responding to a magnetic field and due to the lack of appropriate data for MRE, the B-H curve, shown in Figure 5, which is for the MR fluid with the trade name MRF-132DG from Lord Corporation (Cary, NC, USA) [16], is used in the present work. For the sake of modeling, a second order polynomial, shown by the dashed line in Figure 5, is fitted to the B–H curve that represents the magnetic intensity H (kA/m) as a function of flux density B (T) in the following form:

(2) $$H_{\mathrm{MRE}}=2.89\times 10^{2}\;B^{2}+3.4\times 10\;B$$

**Figure 6:**
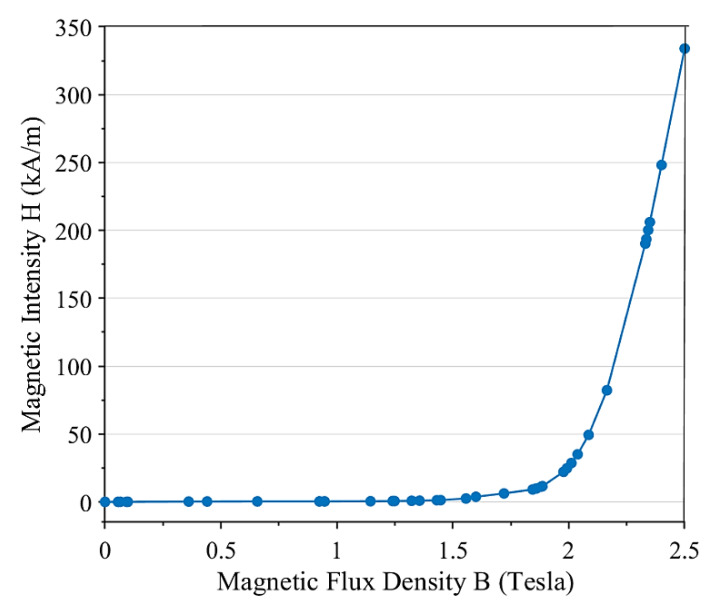
The B–H curve of 1008 steel.

The conductive core of the electromagnet and elastic face layers of the sandwich beam are assumed to be made of 1008 steel material with a density of 7861 kg/m^3^, elastic modulus of 200 GPa, and yield strength of 285 MPa. Data of the B–H curve of this steel type is taken from [17] and is shown in Figure 6. The relationship between the magnetic field intensity H (kA/m) and magnetic flux density B (T) of the 1008 steel is obtained by a curve fitting in [17] with the following polynomial form:

(3) $$H_{\mathrm{steel}}=R_{s0}B^{5}+R_{s1}B^{4}+R_{s2}B^{3}+R_{s3}B^{2}+R_{s4}B+R_{s5}$$

where the coefficients are defined as below:

$$R_{s}=\left\{\begin{array}{cc} \left[0\;\;\;1.82\;\;-3.63\;\;\;1.782\;\;\;0.387\;\;\;0\right] & B\leq 1.5\;T\\ \left[-1419.52\;\;\;13551.37\;\;-50744.31\;\;\;93520.50\;\;-85032.46\;\;\;30566.42\right] & B>1.5\;T \end{array}\right\}$$

In addition, coils of the electromagnets are made of 17 AWG wire with an approximate diameter of D= 1.15 mm [18] and density of 7100 kg/m^3^. With this size, the maximum current that the wire can withstand is expected to be 5 A. The material properties given above are used in the modeling and optimization of the MRE-ATVA in the following sections.

## 4. Finite Element (FE) Modeling of the Sandwich Beam

**Figure 7:**
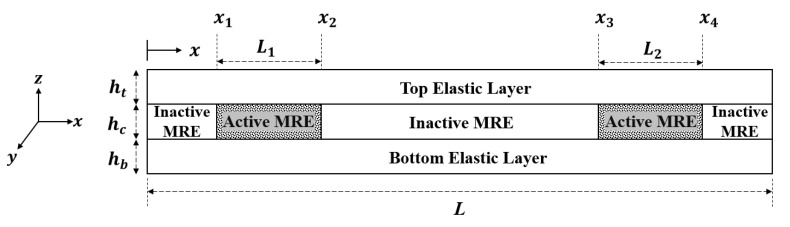
Schematic of the sandwich beam structure with MRE-core layer.

A three-layer sandwich beam with MRE in the core layer is considered with the total length of *L*. The height of each layer and other parameters of the sandwich beam are shown in Figure 7. Since a magnetic field is provided only on specific parts of the MRE layer where the electromagnets are installed, those portions exposed to the magnetic field are activated and the rest of parts are inactive with constant properties. Therefore, the FE model is developed for a sandwich beam with the ability to change the stiffness of only active parts of the MRE core layer. This is similar to the situations where a sandwich beam is partially treated with MRE in the core layer. The location of electromagnets, and thus the position of active parts of MRE could be varied along the beam.

Some logical simplifying assumptions are considered to obtain the governing equations of motion of the sandwich beam through FE analysis [19,20,21]. This includes no slippage between the core layer and the elastic layers, uniform transverse displacement in a given cross-section of the beam, negligible normal stresses in the core layer, and negligible transverse shear strain in the two elastic layers. Using the first order shear deformation theory, the displacement field for each layer at an arbitrary section along the beam span can be expressed as:

(4) $$u_{i}\left(x,z_{i},\;t\right)=u_{i}^{0}\left(x,\;t\right)-z_{i}\frac{\partial w_{i}\left(x,\;t\right)}{\partial x},\;i=t,\;c,\;b$$

(5) $$w_{i}\left(x,\;z_{i},\;t\right)=w\left(x,\;t\right),\;i=t,\;c,\;b$$

where $u_{i}$ is the longitudinal displacement along the x direction and $u_{i}^{0}$ is the displacement of the midplane of the layer which is also the origin of the coordinate $z_{i}$ for each layer; $w_{i}$ is the transverse displacement of each layer equal to the uniform transverse displacement of the beam w; and the subscript i=t, c, b refers to the corresponding layer (t, top layer; c, core layer; and b, bottom layer). The condition of no slippage at the interfaces of the three layers yields the following relation:

(6) $${u_{c}|}_{z_{c}=\frac{h_{c}}{2}}={u_{t}|}_{z_{t}=-\frac{h_{t}}{2}};\;{u_{c}|}_{z_{c}=-\frac{h_{c}}{2}}={u_{b}|}_{z_{b}=\frac{h_{b}}{2}}$$

By substituting Equation (6) into Equations (4) and (5), displacement of the midplane of the core layer can be obtained based on the displacement of the bottom and top layers as:

(7) $$u_{c}^{0}=\frac{u_{t}^{0}+u_{b}^{0}}{2}+\frac{1}{4}\frac{\partial w}{\partial x}\left(h_{t}-h_{b}\right)$$

Movements of the elastic top and bottom layers result in shear deformation in the core layer. The transverse shear strain of the core layer in the x-z plane, thus, can be calculated by taking derivatives of the displacements as:

(8) $$\gamma_{c}^{xz}=\frac{\partial w}{\partial x}+\frac{\partial u_{c}}{\partial z_{c}}$$

in which,

(9) $$\frac{\partial u_{c}}{\partial z_{c}}=\frac{\left(h_{t}+h_{b}\right)}{2h_{c}}\frac{\partial w}{\partial x}+\frac{\left(u_{t}^{0}-u_{b}^{0}\right)}{h_{c}}$$

Thus, using Equation (9), the transverse shear strain in Equation (8) can be described as:

(10) $$\gamma_{c}^{xz}=\frac{D}{h_{c}}\frac{\partial w}{\partial x}+\frac{\left(u_{t}^{0}-u_{b}^{0}\right)}{h_{c}}$$

where $D=h_{c}+\frac{1}{2}\left(h_{t}+h_{b}\right)$. The shear stress in the core layer can be obtained:

(11) $$\tau_{c}^{xz}=G_{c}^{*}\gamma_{c}^{xz}$$

where $G_{c}^{*}$ is the complex shear modulus of the core layer and can be described as follows:

(12) $$G_{c}^{*}=G_{c}^{\prime }+jG_{c}^{\prime \prime }=G_{c}^{\prime }\left(1+j\beta \right)$$

where $G_{c}^{\prime }$ and $G_{c}^{\prime \prime }$ are the storage and loss moduli, respectively, and β is the loss factor of the core layer.

Total strain energy comprises three different parts including bending and extension of the elastic layers and shear deformation of the core layer. The strain energy due to the bending and extension of the top and bottom layers can be written as:

(13) $$V_{t,b}=\frac{1}{2}\int_{0}^{L}\left(E_{t}A_{t}{\left(\frac{\partial u_{t}}{\partial x}\right)}^{2}+E_{b}A_{b}{\left(\frac{\partial u_{b}}{\partial x}\right)}^{2}\right)dx+\frac{1}{2}\int_{0}^{L}\left(E_{t}I_{t}+E_{b}I_{b}\right){\left(\frac{\partial^{2}w}{\partial x^{2}}\right)}^{2}dx$$

where $E_{i}$ is the elastic modulus of the i-th layer (i=t,b); $A_{i}$ is the cross sectional area; and $I_{i}$ the second moment of inertia around the centroid of the layer’s cross section. The strain energy of the viscoelastic core layer is mainly due to the shear deformations, which can be expressed as:

(14) $$V_{c}=\frac{1}{2}\int_{0}^{L}\tau_{c}^{xz}\gamma_{c}^{xz}A_{c}\;dx=\frac{1}{2}\int_{0}^{L}\left(G_{c}^{*}A_{c}\gamma_{c}^{xz}{}^{2}\right)dx=\frac{1}{2}\int_{0}^{L}G_{c}^{*}A_{c}{\left[\frac{D}{h_{c}}\frac{\partial w}{\partial x}+\frac{\left(u_{t}^{0}-u_{b}^{0}\right)}{h_{c}}\right]}^{2}dx$$

Now, the total strain energy of the sandwich beam can be calculated as:

(15) $$V=V_{t,b}+V_{c}$$

Similarly, the kinetic energy is mainly due to the axial and transverse displacements of the elastic and core layers. The kinetic energy associated with the axial displacement of the elastic top and bottom layers ($T_{ax}$) and also due to the transverse displacement of two elastic layers and viscoelastic core layer ($T_{tran}$) can be written as:

(16) $$T_{ax}=\frac{1}{2}\int_{0}^{L}\left(\rho_{t}A_{t}{\left(\frac{\partial u_{t}}{\partial t}\right)}^{2}+\rho_{b}A_{b}{\left(\frac{\partial u_{b}}{\partial t}\right)}^{2}\right)dx$$

and,

(17) $$T_{tran}=\frac{1}{2}\int_{0}^{L}\left(\rho_{t}A_{t}+\rho_{c}A_{c}+\rho_{b}A_{b}\right){\left(\frac{\partial w}{\partial t}\right)}^{2}dx$$

Now, the total kinetic energy can be described as:

(18) $$T=T_{ax}+T_{tran}$$

To construct the FE model of the structure, a one-dimensional two-node sandwich beam element was considered. Each node has four degrees of freedom including the longitudinal displacements of the top and bottom layers ($u_{t},\;u_{b}$), transverse displacement (w), and slope ($\frac{\partial w}{\partial x}=w_{x}$) of the beam. Accordingly, the element nodal displacement vector can be written as:

(19) $$q^{e}\left(t\right)={\left\{q^{1}\;q^{2}\right\}}^{T}={\left\{u_{t}^{1},\;u_{b}^{1},\;w^{1},\;w_{x}^{1},\;u_{t}^{2},\;u_{b}^{2},\;w^{2},\;w_{x}^{2}\right\}}^{T}$$

where $q^{1}={\left\{u_{t}^{1},\;u_{b}^{1},\;w^{1},\;w_{x}^{1}\right\}}^{T}$ and $q^{2}={\left\{u_{t}^{2},\;u_{b}^{2},\;w^{2},\;w_{x}^{2}\right\}}^{T}$ are the nodal displacement vectors at node 1 and 2 of the sandwich beam element, respectively. The longitudinal and transverse displacements of the midplane at each point can be expressed in terms of the elemental displacement vector $q^{e}\left(t\right)$, and shape functions as below:

(20) $$u_{t}^{0}\left(x,\;t\right)=N_{ut}\left(x\right)q^{e}\left(t\right)\;u_{b}^{0}\left(x,t\right)=N_{ub}\left(x\right)q^{e}\left(t\right)\;w\left(x,t\right)=N_{w}\left(x\right)q^{e}\left(t\right)$$

Here we have utilized linear shape function for the longitudinal displacement and cubic shape function for the transverse displacement as:

(21) $$N_{ut}\left(x\right)=\left[\left(1-\frac{x}{L_{e}}\right),\;0\;,\;0,\;0,\left(\frac{x}{L_{e}}\right),0,\;0,\;0\right]\;N_{ub}\left(x\right)=\left[0,\left(1-\frac{x}{L_{e}}\right),0,\;0,\;0,\;\left(\frac{x}{L_{e}}\right),0,\;0\right]\;N_{w}\left(x\right)=\left[0,\;0,\;\left(1-\frac{3x^{2}}{L_{e}^{2}}+\frac{2x^{3}}{L_{e}^{3}}\right),\left(x-\frac{2x^{2}}{L_{e}}+\frac{x^{3}}{L_{e}^{2}}\right),0,\;0,\;\left(\frac{3x^{2}}{L_{e}^{2}}-\frac{2x^{3}}{L_{e}^{3}}\right),\left(-\frac{x^{2}}{L_{e}}+\frac{x^{3}}{L_{e}^{2}}\right)\right],$$

where $L_{e}$ is length of each element. Substituting Equations (20) and (21) into Equations (4) and (5), and then by substituting the resulting displacement field into energy relations, the potential energy of the element can be found using Equation (15) in the matrix form with respect to the element nodal displacement vector as:

(22) $$V=\frac{1}{2}{\left\{q^{e}\right\}}^{T}\left(\left[K_{1}\right]+\left[K_{2}\right]+\left[K_{3}\right]+\left[K_{4}\right]\right)\left\{q^{e}\right\}$$

where $\left[K_{i}\right],\;i=1,\;2,\;3,\;4$ are obtained as:

(23) $$\left[K_{1}\right]=\int_{0}^{L_{e}}\left(E_{t}A_{t}{\left[\frac{dN_{ut}}{dx}\right]}^{T}\left[\frac{dN_{ut}}{dx}\right]+E_{b}A_{b}{\left[\frac{dN_{ub}}{dx}\right]}^{T}\left[\frac{dN_{ub}}{dx}\right]\right)dx\;\left[K_{2}\right]=\int_{0}^{L_{e}}\left(\frac{h_{t}^{2}}{12}+\frac{h_{b}^{2}}{12}\right){\left[\frac{d^{2}N_{w}}{dx^{2}}\right]}^{T}\left[\frac{d^{2}N_{w}}{dx^{2}}\right]dx\;\left[K_{3}\right]=\int_{0}^{L_{e}}\left(E_{t}I_{t}+E_{b}I_{b}\right){\left[\frac{d^{2}N_{w}}{dx^{2}}\right]}^{T}\left[\frac{d^{2}N_{w}}{dx^{2}}\right]dx\;\left[K_{4}\right]=\int_{0}^{L_{e}}G_{c}^{*}A_{c}{\left[\frac{D}{h_{c}}\left[\frac{dN_{w}}{dx}\right]+\left(\frac{\left[N_{ut}\right]-\left[N_{ub}\right]}{h_{c}}\right)\right]}^{T}\left[\frac{D}{h_{c}}\left[\frac{dN_{w}}{dx}\right]+\left(\frac{\left[N_{ut}\right]-\left[N_{ub}\right]}{h_{c}}\right)\right]dx$$

Similarly, the kinetic energy of the element by using Equation (18) yields the following equations:

(24) $$T=\frac{1}{2}{\left\{{\dot{q}}^{e}\right\}}^{T}\left(\left[M_{1}\right]+\left[M_{2}\right]+\left[M_{3}\right]+\left[M_{4}\right]\right)\left\{{\dot{q}}^{e}\right\}$$

where $\left[M_{i}\right],\;i=1,\;2,\;3,\;4$ are defined as:

(25) $$\left[M_{1}\right]=\int_{0}^{L_{e}}\left(\rho_{t}A_{t}{\left[N_{ut}\right]}^{T}\left[N_{ut}\right]+\rho_{b}A_{b}{\left[N_{ub}\right]}^{T}\left[N_{ub}\right]\right)dx\;\left[M_{2}\right]=\int_{0}^{L_{e}}\left(\rho_{t}A_{t}+\rho_{c}A_{c}+\rho_{b}A_{b}\right){\left[N_{w}\right]}^{T}\left[N_{w}\right]dx\;\left[M_{3}\right]=\int_{0}^{L_{e}}\frac{1}{12}\left(\rho_{t}A_{t}h_{t}^{2}{\left[\frac{dN_{w}}{dx}\right]}^{T}\left[\frac{dN_{w}}{dx}\right]+\rho_{b}A_{b}h_{b}^{2}{\left[\frac{dN_{w}}{dx}\right]}^{T}\left[\frac{dN_{w}}{dx}\right]\right)dx\;\left[M_{4}\right]=\int_{0}^{L_{e}}\frac{\rho_{c}A_{c}h_{c}^{2}}{12}{\left[\frac{D}{h_{c}}\left[\frac{dN_{w}}{dx}\right]+\left(\frac{\left[N_{ut}\right]-\left[N_{ub}\right]}{h_{c}}\right)\right]}^{T}\left[\frac{D}{h_{c}}\left[\frac{dN_{w}}{dx}\right]+\left(\frac{\left[N_{ut}\right]-\left[N_{ub}\right]}{h_{c}}\right)\right]dx$$

Finally, the governing equations of motion can be obtained using Lagrange’s energy method:

(26) $$\frac{d}{dt}\left(\frac{\partial T}{\partial \dot{q_{i}}}\right)-\frac{\partial T}{\partial q_{i}}+\frac{\partial V}{\partial q_{i}}=F_{i},\;i=1,\ldots ,\;n$$

where $q_{i}$ is the i-th degree of freedom of an element, $F_{i}$ is the generalized force corresponding to the i-th degree of freedom, and n is the total number of degrees of freedom of the element. By using energy terms in matrix forms in Equations (22) and (24) into Lagrange’s equations, the governing equations of motion for the sandwich beam element in FE form can be written as:

(27) $$\left[M^{e}\right]\left\{\ddot{q_{e}}\right\}+\left[K^{e}\right]\left\{q^{e}\right\}=\left\{F^{e}\right\}$$

where $\left[M^{e}\right]=\left[M_{1}\right]+\left[M_{2}\right]+\left[M_{3}\right]+\left[M_{4}\right]$ is the element mass matrix and $\left[K^{e}\right]=\left[K_{1}\right]+\left[K_{2}\right]+\left[K_{3}\right]+\left[K_{4}\right]$ is the element stiffness matrix. $\left\{q^{e}\right\}$ and $\left\{\ddot{q_{e}}\right\}$ are the vectors of nodal displacements and nodal accelerations, respectively, and $\left\{F^{e}\right\}$ is the elemental force vector. By assembling all the elemental mass and stiffness matrices of the structure, the system governing equations of motion can be obtained as:

(28) $$\left[M\right]\left\{\ddot{Q}\right\}+\left[K\right]\left\{Q\right\}=\left\{F\right\}$$

where [M] is the system mass matrix, [K] is the system stiffness matrix, {Q} and $\left\{\ddot{Q}\right\}$ are the system vectors of nodal displacements and accelerations, respectively, and {F} is the system load vector. Respecting partial or full treatment of the sandwich beam with MRE in the core layer, the assembly of the elemental matrices is performed accordingly in the developed FE code.

To find the natural frequency and loss factor of the sandwich beam, the problem of free vibration is studied considering $\left\{F\right\}=\vec{0}$ in Equation (28). Then, by solving the resulting eigenvalue problem, the eigenvalues of the system can be calculated. The square root of the real part of the eigenvalues provides the natural frequencies of the system, whereas the ratio of the imaginary part to the real part of the eigenvalues yields the loss factor. The developed FE model of the sandwich beam is programmed in a MATLAB^®^ environment (R2019a, MathWorks Inc., Natick, MA, USA).

### Validation of the Developed FE Model

**Figure 8:**
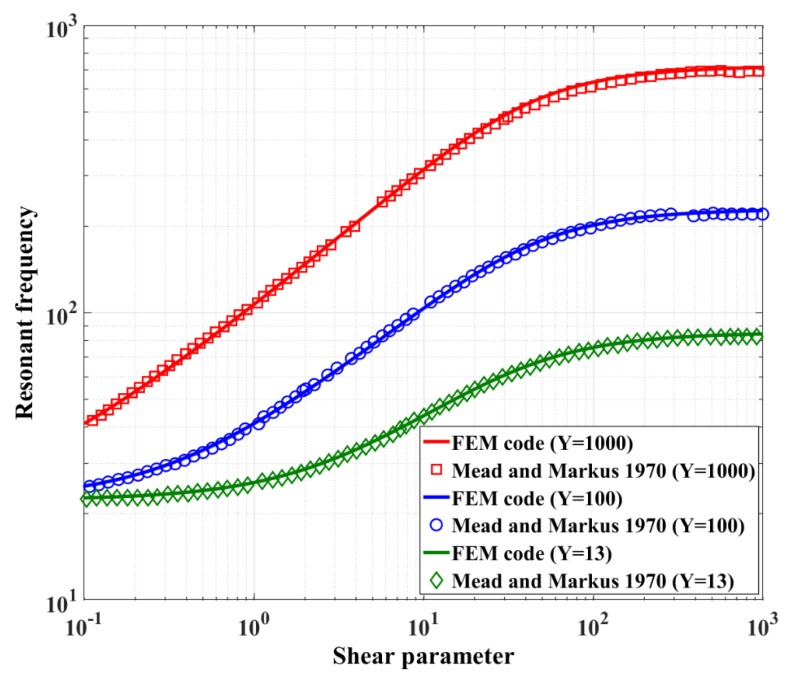
Comparison of the results of the developed finite element (FE) model with analytical results of Mead and Markus 1970 [22].

The developed FE model of the sandwich beam was validated through a comparison of the present results with those reported by Mead and Markus [22] using an analytical approach for a clamped-clamped sandwich beam with two elastic face layers and a viscoelastic core layer. Figure 8 shows the results for the resonant frequency of the sandwich beam with respect to the dimensionless shear parameter which is defined by Equations (3a) and (3b) in page 101 of [22]. The results are shown for three different values of the geometric parameter Y as defined in Equation (4a) in [22] and the loss factor of the core layer is set at β = 1. As shown in the figure, it can be realized that excellent agreement exists between the developed FE model and the analytical results.

## 5. Magnetostatic Modeling and Analysis of the Electromagnets

The shear modulus of the active portions of the MRE core layer depends on the applied magnetic field by the electromagnets. Here, the analytical magnetic circuit model for different proposed configurations of electromagnets is presented, and then validated using magnetostatic FE analysis. Then, the analytical models were effectively utilized to evaluate the magnetic flux density which was applied at the center of the gap where the sandwich beam was located for the given applied current and magnetic circuit parameters.

Two fundamental laws governing the performance of the electromagnets are the Ampere’s and Gauss’s laws that involve magnetic field and magnetic flux, respectively. The Ampere’s circuit law states that the line integral of the magnetic field intensity over any closed path is equal to the net current enclosed by that path which can be mathematically expressed as [23]:

(29) $$\oint_{C}\vec{H}.\vec{dl}=I_{enc}$$

where $\vec{H}$ is the vector of the magnetic field intensity, $\vec{dl}$ is an incremental segment of the closed path C, and $I_{enc}$ is the total current flowing through the surface that is enclosed by the closed path. In Equation (29), the sign of $I_{enc}$ is determined by the right-hand rule, however, the Gauss’s law states that over any closed volume, the surface integral of flux density is zero which can be expressed as:

(30) $$\oint_{S}\vec{B}.\vec{dA}=0$$

where $\vec{B}$ is magnetic flux density and $\vec{dA}$ is an infinitesimal element of the closed surface S which encloses the volume. Generally, it is a complex problem to solve Equations (29) and (30) because of the different sections in the electromagnet, as well as the variation of magnetic parameters. However, in the electromagnet devices, we only need the magnetic field at the center of the gap where the MRE core layer is located.

As a simplifying assumption, we consider that the magnetic field is constant for specific parts along the center line of the conductive core of each electromagnet (dashed lines in Figure 2). As a result, we can write Equation (29) in a discretized form by replacing the integration with summation which is basically the analog of Kirchhoff’s voltages law for magnetic circuits and can be expressed as:

(31) $$\sum H_{i}l_{i}=\sum N_{i}I_{i}$$

where $H_{i}$ is magnetic field in the i-th portion along the core’s center line with constant magnetic flux. The parameters $l_{i}$, $N_{i}$, and $I_{i}$ stand for the length of the portion, number of turns of wire included, and input current to the coil, respectively. On the one hand, Equation (31) states that the sum of magnetomotive force drops ($H_{i}l_{i}$) around a closed loop is equal to the sum of the magnetomotive force sources ($N_{i}I_{i}$) in that loop.

On the other hand, Equation (30) states that the sum of the fluxes (ϕ) flowing into any closed volume in the space must be zero. Considering a node (very small volume) in the path of the magnetic flux flow, then, the sum of the fluxes into or out of the node must be zero which is the analog of Kirchhoff’s current law for magnetic circuits and may be mathematically stated as:

(32) $$\sum \phi_{i}=0$$

**Figure 9:**
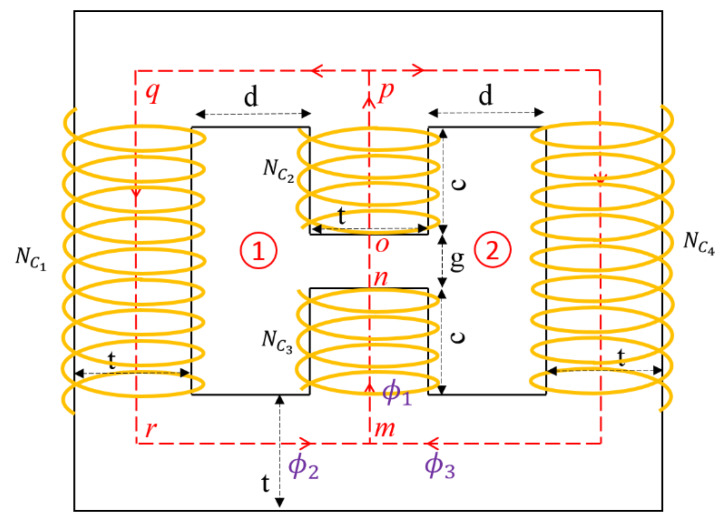
The H-shaped electromagnet with the path of magnetic flux penetration discretized by letters for magnetic analysis.

Equations (31) and (32) are used in the analysis of the electromagnets. Here, the development of the analytical circuit model is presented for the H-shaped electromagnet, as shown in Figure 9. The path of the magnetic flux which flows through core sections is shown in the figure with a dashed line.

There are two closed loops for the H-shaped electromagnet, as shown in Figure 9. For the closed loop number 1, starting from point *n* and moving in a counterclockwise direction along the path *nopqrmn*, Equation (31) can be expanded over different segments along the path as:

(33) $$N_{c1}I+N_{c2}I+N_{c3}I=H_{core}{}_{op}l_{op}+H_{core}{}_{pqrm}l_{pqrm}+H_{core}{}_{mn}l_{mn}+H_{gap}l_{gap}$$

where I is the input current to the coils, and $N_{c_{i}}$ (i=1, 2, 3, 4) is the number of turns of wire in the $C_{i}$, i.e., coil of the electromagnet. Magnetic field (H) and length (l) for each segment are shown with corresponding subscripts. The magnetic flux density is assumed to be constant at any cross-section along the core’s centerline, i.e., ϕ=BA. Moreover, at juncture points *m* or *p*, considering Equation (32), we can write:

(34) $$\phi_{1}=\phi_{2}+\phi_{3}$$

in which $\frac{\phi_{1}}{2}=\phi_{2}=\phi_{3}$ due to the symmetry of the electromagnet. The same relation exists for the magnetic flux density regarding the cross-section is the same all around the closed path.

According to the geometrical parameters in Figure 2a and Figure 9, the number of turns of wire in the coils of the electromagnet can be determined as below:

(35) $$N_{c1}=N_{c4}=\frac{d\left(2c+g\right)}{2D^{2}}\;N_{c2}=N_{c3}=\frac{cd}{2D^{2}}$$

where c, d, and g are geometric parameters shown in Figure 9 and D is the wire diameter. In addition, the total mass of the electromagnet can be formulated as:

(36) $$M_{el}=\frac{\pi }{4}D^{2}L_{w}\rho_{w}+V_{sc}\rho_{sc}$$

where $\rho_{w}$ and $\rho_{sc}$ are densities of the wire and steel core of the electromagnet. $L_{w}$ is the length of wire and can be found with the following equation:

(37) $$L_{w}=\frac{2d\left(2c+g\right)}{D^{2}}\left(t+t+d\right)+\frac{2cd}{D^{2}}\left(t+t+d\right)$$

and $V_{sc}$ is the volume of the steel core and can be expressed as:

(38) $$V_{sc}=t\left(\left(2d+3t\right)\left(2t+2c+g\right)-2d\left(2c+g\right)-gt\right)$$

Using Equations (33) to (38), the H-shaped electromagnet can be analyzed to find the magnetic flux density at the center of the gap when the input current to the coil is known.

**Figure 10:**
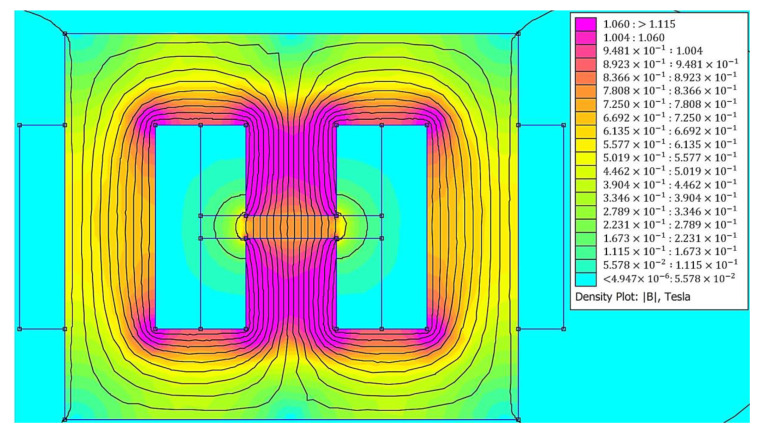
Magnetic analysis of the considered H-shaped electromagnet using FEMM software.

Here, the results for the analytical magnetic circuit model developed above is compared with the obtained results using magnetostatic FE analysis. For this purpose, the H-shaped electromagnet with dimensions of c=d=t= 2 cm and a gap of g= 0.5 cm is considered. Figure 10 shows the FE magnetic analysis performed by the FEMM software, which is a popular open source FE-based software for magnetic analysis. Here, the input current to the coil is set at 5 A.

The magnetic flux density at the center of the gap has been evaluated using both analytical and FE models for different applied currents and the results are provided in Table 1. As it is observed, although the presented analytical formulation does not consider the phenomena of fringing and leakage of the magnetic flux, very good agreement exists between analytical and FE results.

**Figure A1:**
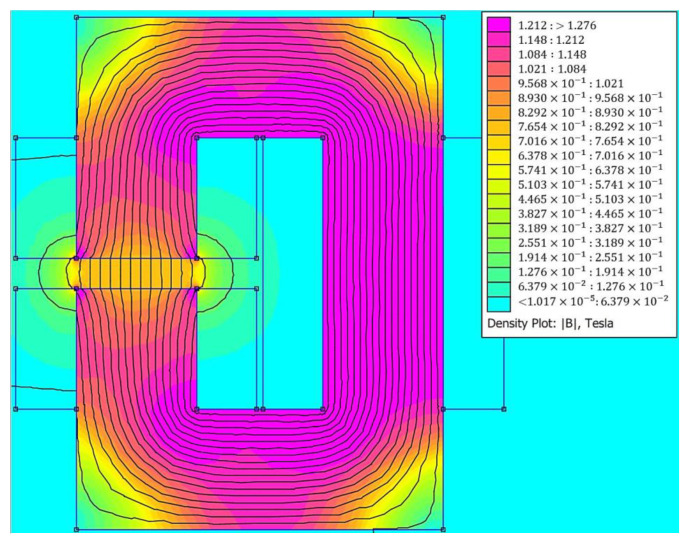
Magnetic flux distribution for the C-shaped electromagnet using FEMM software.

**Figure A2:**
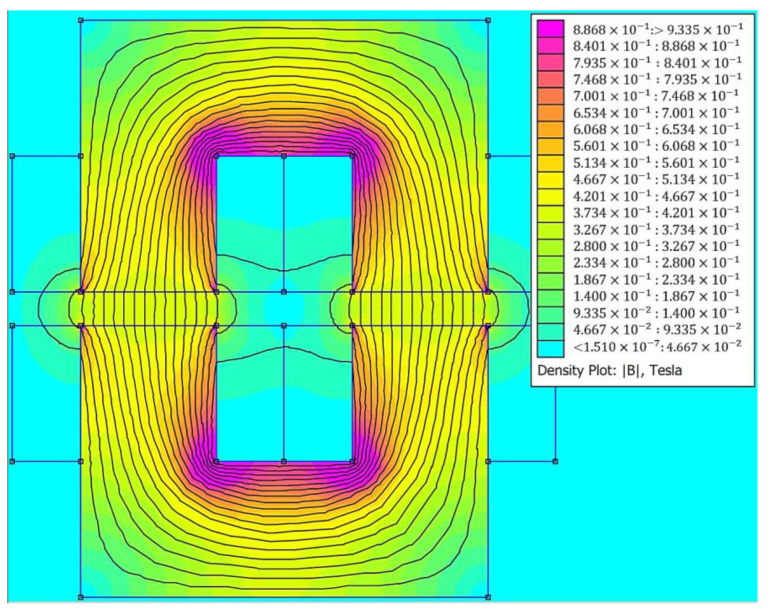
Magnetic flux distribution for the U-shaped electromagnet using FEMM software.

It should be noted that a similar analysis can be conducted for the C- and U-shaped electromagnets. The formulation for the C- and U-shaped electromagnets are provided in Appendix A. (FE magnetic analysis of the C-shaped and U-shaped electromagnets are shown in Figure A1 and Figure A2, respectively. Comparison of flux density at the center of the air gap of the C-shaped and U-shaped electromagnets are also provided in Table A1 and Table A2, respectively). Similar to the H-shaped electromagnet, negligible error has been observed between analytical and FE magnetic circuit results for the C- and U-shaped electromagnets.

By combining the developed FE model for the MRE-based sandwich beam with the analytical magnetic circuit model of the electromagnet, a complete model of the MRE-ATVA is obtained. Then, this high-fidelity model was utilized to evaluate the objective function (natural frequency of the MRE-ATVA) and constraint functions (total weight of the MRE-ATVA as well as the static deflection and stresses experienced in the sandwich beam due to the weight of the electromagnet) required for the formulation of the multidisciplinary design optimization problem.

## 6. Multidisciplinary Design Optimization of the Proposed MRE-Based ATVAs

In this study, a multidisciplinary design optimization problem involving the electromagnet and sandwich beam structure design parameters was formulated to maximize the frequency bandwidth of the proposed MRE-ATVA under mass, deflection, and stress constraints. Due to the symmetry in the proposed configurations for the MRE-ATVA, as shown in Figure 3, half of the absorber structure, which is a cantilever MRE-sandwich beam with one electromagnet installed at the free end, was considered. The active part of the MRE layer (see Figure 7) is at the free end of the sandwich beam where the electromagnet is installed. The design optimization was conducted on different design configurations of the electromagnets (H-, C-, and U-shaped) to identify the optimal configuration.

There are several important practical factors in the design optimization of the proposed MRE-ATVA such as weight, operating frequency range, static tip deflection of the cantilever beam, and maximum stress in the elastic layers of the beam. In the proposed optimization problem, the objective is to maximize the frequency bandwidth of the proposed MRE-ATVA under mass, deflection, and stress constraints. In order to realize a light weight MRE-ATVA, the total mass of the device is considered to be less than 700 g. Moreover, to avoid large deflections that can cause geometric nonlinearities, the static tip deflection of the beam is expected to be not more than 10% of the beam length. Additionally, the maximum stress in the elastic face layers which occurs at the root of the cantilever sandwich beam should not surpass the yield strength of the material. Finally, since the proposed ATVA is considered for vibration control applications in the low frequency ranges, the absorber’s frequency in the off-state condition, i.e., the natural frequency under zero current in the coils (electromagnets are off), is restricted to be less than 10 Hz.

Figure 2 and Figure 7 show all the important geometric dimensions in the configuration of the electromagnets and sandwich beam, respectively. The parameters of the core of the electromagnets, c and d, and the thickness of the beam layers $h_{t}$, $h_{c}$, and $h_{b}$ are identified as the important design variables in the optimization problem. By having a symmetric sandwich beam with equal thickness in the top and bottom elastic layers, i.e., $h_{t}=h_{b}$, there are four design variables ($c\;,\;d,\;h_{t}\;and,\;h_{c}$) in the optimal design problem. It should be noted that the gap of the electromagnet, g, is occupied by the sandwich beam, and thus it would be a dependent parameter ($g=2h_{t}+h_{c}$). The other independent parameters and dimensions are assumed to be fixed. In summary, the design optimization problem of the proposed MRE-ATVA can be formally formulated as:

Find the design variables: $\left[c,\;d,\;h_{t},\;h_{c}\right]$

(39) $$\mathrm{To}\;\mathrm{minimize}:\;\left[\frac{{\left(f\right)}_{B_{1}=0}}{{\left(f\right)}_{B_{2}}}\right]$$

Subject to the following behavior constraints:

(40) $$M_{total}\leq 700\;g$$

(41) $${\left(f\right)}_{B_{1}=0}\leq 10\;\mathrm{Hz}$$

(42) maximum stress in the elastic layers≤285 MPa

(43) static tip deflection≤0.1 L

and side constraints,

(44) $$\left[c_{\min},\;d_{\min},\;h_{t}{}_{\min},\;h_{c}{}_{\min}\right]\leq \left[c,\;d,\;h_{t},\;h_{c}\right]\leq \left[c_{\max},\;d_{\max},\;h_{t}{}_{\max},\;h_{c}{}_{\max}\right]$$

where $M_{total}$ is the total mass of the ATVA which is the sum of the sandwich beam mass and the electromagnet mass; ${\left(f\right)}_{B_{i}}$ i=1,2 is the natural frequency of the ATVA when flux density of $B_{i}$ is applied on the MRE layer by the electromagnet; and $B_{1}=0$ is when the electromagnet is off and $B_{2}$ is the flux density that is generated by the electromagnet when maximum allowable current is supplied to the coils. Minimizing the ratio of $\left[\frac{{\left(f\right)}_{B_{1}=0}}{{\left(f\right)}_{B_{2}}}\right]$, infers the lowest value for ${\left(f\right)}_{B_{1}=0}$ and the highest value for ${\left(f\right)}_{B_{2}}$, which is indeed maximization of the frequency range of the ATVA. The design variables are bounded by reasonable lower and upper limits. In the present study, the values for the lower limit are as follows: $c_{\min}=d_{\min}=5\;\mathrm{mm}$, $h_{t}{}_{\min}=h_{c}{}_{\min}=0.5\;\mathrm{mm}$, and for the upper limit, $c_{\max}=d_{\max}=30\;\mathrm{mm}$; $h_{t}{}_{\max}=h_{c}{}_{\max}=3$ mm.

In the present study, the combination of genetic algorithm (GA) and sequential quadratic programming (SQP) method is utilized to accurately catch the true optimum solution. The GA is a random-based evolutionary algorithm which can approximately identify the global optimum solution and SQP is a powerful derivative-based algorithm developed for constrained nonlinear optimization problems. Depending on the starting initial point, SQP can accurately identify the nearest local optimum solution, without any mechanism to search for global solution. In this study, the merits of both algorithms have been exploited in which the GA is first run to identify the near global optimum solution and then the optimum solution from GA is fed into the SQP algorithm as the initial point to capture accurately the global optimum solution.

The complete model of the ATVA, which consists of the FE model of the sandwich beam and magnetic analysis of the electromagnet, is used to develop two MATLAB^®^ functions; one function to calculate the objective function defined in Equation (39) and the other function includes constraints defined in Equations (40) to (44). The optimization problem for each configuration of the electromagnet is first solved by the genetic algorithm using the GA optimization platform in MATLAB^®^. Then, the optimal result of GA is used as the initial point to run the “Fmincon” command with the SQP algorithm using MATLAB^®^.

## 7. Optimization Results and Discussion

In this section, the optimum results for different configurations of the MRE-ATVA are presented and compared. In all cases, the length and width of the three-layer sandwich beam is fixed to be L = 15 cm and w = 2 cm, respectively. The edge size of the square cross-section of the conductor core is set at t=2 cm for all the electromagnet designs. The maximum applied current is considered to be 5 A. The material properties for different parts of the ATVA and their mechanical and magnetic properties were introduced in Section 3.

**Figure 11:**
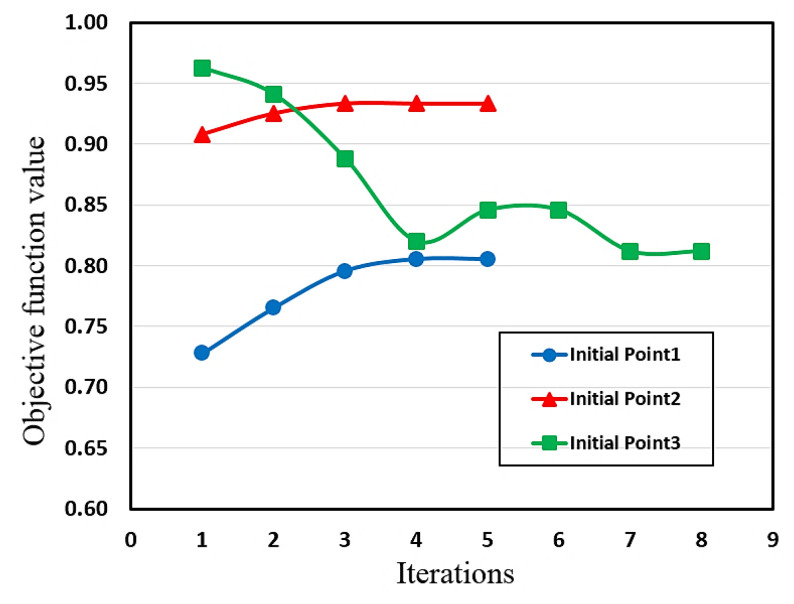
Performance of the sequential quadratic programming (SQP) method when starting from different initial points.

First, to demonstrate the non-convexity nature of the problem and existence of multiple local optimum solutions, the SQP optimizer was executed for MRE-ATVA with H-shaped electromagnet using different initial points. Figure 11 shows the iteration history (objective function versus number of iterations). The three initial points were selected from the beginning, middle, and end of the design variables’ range. As shown in Figure 11, it can be realized that starting from different initial points, the SQP method identifies different local optimum solutions.

Executing GA, which uses random population of points, resulted in optimum solutions which were close to the global optimum solution. However, once different optimum solutions from the GA were used as initial points for the SQP algorithm, a unique global optimum solution was obtained.

Table 2 shows the optimization results for different configurations of the proposed MRE-ATVA with a combination of the GA and SQP methods. The optimal values of the design variables are reported on the top part of the table which are used to calculate the corresponding performance of the optimal MRE-ATVAs provided at the bottom of the table. The results suggest that the optimal MRE-ATVA with U-shaped electromagnet presents a 42.1% frequency increase, whereas the C- and H-shaped types show a 28% and 24% increase in the frequency, respectively. Therefore, the ATVA having a U-shaped electromagnet shows the maximum frequency range as compared with the other design configurations where the natural frequency of the absorber could vary from 5.73 to 8.14 Hz. In addition, the optimal C-shaped design shows the minimum mass of 423 g, while the U- and H-shaped designs give a mass of 596 and 643 g, respectively.

The reason behind a higher frequency bandwidth of the optimal U-shaped configuration over the C- and H-shaped designs could be due to the wider interaction surface of the U-shaped electromagnet with the MRE core layer. This type of electromagnet, due to its U-shaped structure, has two contact surfaces on its poles with the sandwich beam where the magnetic field flows through the MRE core layer. Therefore, the MRE layer is activated in a wider span of the sandwich beam which causes a higher MR effect in the system, and finally greater change in the natural frequency. Moreover, it should be noted that the constraint of maximum stress in the steel face layers, at the root of the beam, is an active constraint for the optimal design of all three ATVA configurations presented in Table 2. As seen from the results in Table 2, the optimal ATVA with the U-shaped electromagnet, thanks to its location on the beam with a center of mass closer to the beam root, allows for a thicker MRE layer (higher $h_{c}$) before reaching the limit of maximum stress. A thicker MRE core layer results in a higher MR effect in the system which results in a greater change in the natural frequency.

**Figure 12:**
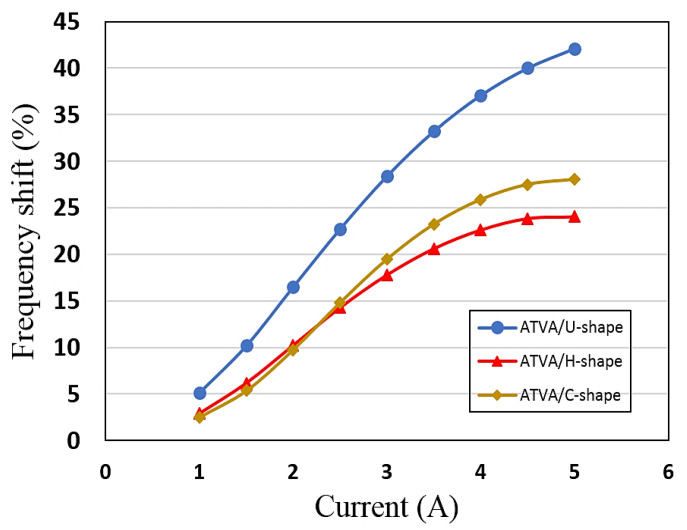
Frequency shift versus input current for the three optimal MRE-ATVAs.

Figure 12 illustrates the optimal frequency shift for the MRE-ATVAs with different values of the input current to the coils of the electromagnets. As shown in the figure, it can be realized, on the one hand, that the optimal ATVA having U-shaped electromagnet has the highest frequency shift for all current inputs as compared with those of C- and H-shaped designs. On the other hand, comparison of the C- and H-shaped configurations shows that they have a similar performance at the low current values, whereas for the current values more than 2.5 A, the C-shaped design precedes the H-shaped configuration. For all three configurations, the slope of the graphs is greater at small current values and gradually decreases with an increase of the input current which can be attributed to the magnetic saturation of the MRE at high values of magnetic excitation, as discussed in Section 3.

## 8. Conclusions

In this study, a comprehensive design optimization process for a novel MRE-based adaptive tuned vibration absorber (MRE-ATVA) is investigated. The proposed MRE-ATVA consists of a three-layer sandwich beam with MRE in the core layer and two electromagnets placed on both ends of the beam to provide the required magnetic field and to serve as the active mass of the absorbers. Three potential designs for the electromagnets are investigated in the optimization problem including electromagnets with U-, H-, and C-shaped designs. A finite element (FE) model of the sandwich beam is presented and validated with the analytical solution from the literature. Magnetic analysis of the electromagnets is performed using a developed formulation based on the Ampere’s circuital law and Gauss’s law. The results of the magnetic analysis of the electromagnets are validated with the simulation results using a FE magnetic analysis. By combining the FE model of the sandwich beam and the magnetic model of the electromagnets, a high-fidelity model of the proposed MRE-ATVAs is constructed, which is subsequently employed to calculate the natural frequency of the complete absorber device and constraint functions.

A multidisciplinary design optimization problem is finally formulated to identify the optimal values of the considered geometric structural and magnetic design parameters to maximize the frequency range of the proposed MRE-ATVA. A combined GA and SQP algorithm was used to find accurate global optimal solutions of the proposed MRE-ATVAs with three different electromagnet configurations. The results suggest that the optimal MRE-ATVA with U-shaped electromagnet can provide the highest frequency shift (42%) in the range of 5.73 to 8.14 Hz. The C- and H-shaped configurations present a 28% and 24% increase in the natural frequency, respectively. The optimal adaptive tuned vibration absorbers are all lightweight as their mass is below 700 g. In addition, the performance of the optimized MRE-ATVA at different input currents is investigated. The results show that the optimal MRE-ATVA with a U-shaped electromagnet provides the highest frequency shift irrespective of the applied current. Such optimized lightweight MRE-ATVAs with significant frequency shift in the low frequency range paves the way for realization of the compact practical adaptive vibration absorbers that are very effective and reliable for vibration and noise control applications.

## Figures and Tables

**Table 1:** Comparison of flux density values at the center of the air gap of the H-shaped electromagnet obtained by analytical magnetic circuit model (Equations (33) to (38)) and FE model.

Input Current (A)	Flux Density (mT) Analytical Magnetic Circuit Model Equations (33) to (38)	Flux Density (mT) FE Analysis	Error %
2.5	399	402	0.77
3	479	482	0.73
3.5	559	563	0.71
4	640	643	0.53
4.5	720	724	0.55
5	801	804	0.37

**Table 2:** Optimization results of the MRE-ATVA with the three different electromagnets.

Parameter		ATVA with U-shaped Electromagnet 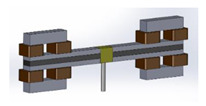	ATVA with H-shaped Electromagnet 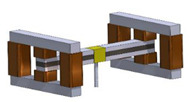	ATVA with C-shaped Electromagnet 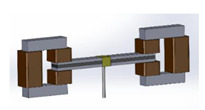
**Optimal Values (mm)**	$h_{t}$ **=** $h_{b}$	0.85	0.95	0.79
	$h_{c}$	1.10	0.50	0.50
	c	5.00	5.00	5.00
	d	18.00	5.00	5.00
**ATVA Performance**	**Total Mass (g)**	596.0	643.0	423.0
	**Frequency Range [** $f_{1}$ **−** $f_{2}$ **] (Hz)**	5.73–8.14	5.38–6.68	5.23–6.69
	Frequency Shift Δf **(Hz)**	2.41	1.30	1.46
	**Frequency Shift** $\frac{\Delta f}{f_{1}}$ **(%)**	42.1	24.0	28.0

## Appendix A

In this appendix, required formulation for magnetic analysis of the C- and U-shaped electromagnets is provided, similarly, to the equations developed for the H-shaped electromagnet in Section 5. Parameters have the same meaning as in the case of H-shaped electromagnet unless otherwise mentioned.

For the C-shaped electromagnet, according to Figure 2b that shows the C-shaped electromagnet, since there is only one closed loop in the path of magnetic flux without any node, the amount of flux is constant around the loop. In addition, Equation (31) could be written for the C-shaped electromagnet in the following format:

(A1) $$N_{c1}I+N_{c2}I+N_{c3}I=H_{core}l_{core}+H_{gap}l_{gap}$$

The number of turns of wire in the coils of the electromagnet can be estimated as:

(A2) $$N_{c1}=N_{c2}=\frac{cd}{2D^{2}}\;N_{c3}=\frac{d\left(2c+g\right)}{2D^{2}}$$

In addition, the total mass of the electromagnet can be formulated as:

(A3) $$M_{el}=\frac{\pi }{4}D^{2}L_{w}\rho_{w}+V_{sc}\rho_{sc}$$

where $L_{w}$ and $V_{sc}$ could be found with the following relations:

(A4) $$L_{w}=\frac{2cd}{D^{2}}\left(t+t+d\right)+\frac{d\left(2c+g\right)}{D^{2}}\left(t+t+d\right)$$

(A5) $$V_{sc}=t\left(\left(2t+d\right)\left(2t+2c+g\right)-d\left(2c+g\right)-gt\right)$$

Similar to what was presented for the H-shaped electromagnet in Figure 10, here, the FE magnetic analysis for the C-shaped electromagnet, performed by the FEMM software, is demonstrated in Figure A1. The input current to the coil is set at 5 A. The considered C-shaped electromagnet has dimensions of c=d=t=2 cm and a gap of g= 0.5 cm.

Results of magnetic flux density at the center of the gap has also been calculated using both analytical model (Equations (A1) to (A5)) and FE analysis (with FEMM software) for different applied currents. Table A1 shows the comparison of the results for the C-shaped electromagnet.

**Table A1:** Comparison of flux density values at the center of the air gap of the C-shaped electromagnet obtained by analytical magnetic circuit model (Equations (A1) to (A5)) and FE model.

Input Current (A)	Flux Density (mT) Analytical Magnetic Circuit Model Equations (A1) to (A5)	Flux Density (mT) FE Analysis	Error%
2.5	394	400	1.50
3	474	480	1.25
3.5	554	560	1.07
4	635	645	1.55
4.5	716	726	1.38
5	798	806	0.99

For the U-shaped design, considering the U-shaped electromagnet as in Figure 2c, the analytical magnetic model can be presented with the following equations:

(A6) $$N_{c1}I+N_{c2}I+N_{c3}I+N_{c4}I=H_{core}l_{core}+H_{gap}l_{gap}$$

The number of turns of wire in the coils of the electromagnet can be found as:

(A7) $$N_{c1}=N_{c2}=N_{c3}=N_{c4}=\frac{cd}{2D^{2}}$$

Moreover, the total mass of the electromagnet can be formulated as:

(A8) $$M_{el}=\frac{\pi }{4}D^{2}L_{w}\rho_{w}+V_{sc}\rho_{sc}$$

where length of wire $L_{w}$ and volume of the steel core $V_{sc}$ could be found with the following relations:

(A9) $$L_{w}=\frac{4cd}{D^{2}}\left(t+t+d\right)$$

(A10) $$V_{sc}=2t\left(\left(2t+d\right)\left(c+t\right)-cd\right)$$

Figure A2 shows the FE magnetic analysis for the U-shaped electromagnet, performed by the FEMM software when the input current to the coil is set at 5 A.

In addition, the results of magnetic flux density at the center of the gap calculated by utilizing both analytical model (using Equations (A6) to (A10)) and FE analysis (with FEMM software) for different input currents are shown in Table A2 for the U-shaped electromagnet.

**Table A2:** Comparison of the flux density values at the center of the air gap of the U-shaped electromagnet obtained by analytical magnetic circuit model (Equations (A6) to (A10)) and FE model.

Input Current (A)	Flux Density (mT) Analytical Magnetic Circuit Model Equations (A6) to (A10)	Flux Density (mT) FE Analysis	Error%
2.5	189	190	0.53
3	226	228	0.88
3.5	264	266	0.75
4	302	304	0.66
4.5	339	341	0.59
5	377	379	0.53
